# Split Potassium Fertilization Modulates Endogenous Hormone Homeostasis to Optimize the Grain-Filling Process and Mitigate High-Temperature Damage in Rice

**DOI:** 10.3390/plants15121781

**Published:** 2026-06-09

**Authors:** Xinyue Zhang, Junjie Dong, Youfa Li, Yuanze Sun, Haowei Fu

**Affiliations:** 1 Jiaxing Academy of Agricultural Sciences, Jiaxing 314016, China; drzhangxinyue@163.com (X.Z.); junjie.dong123@outlook.com (J.D.); liyoufa66@sina.com (Y.L.); 2 Liaoyuan Academy of Agricultural Sciences, Liaoyuan 136299, China; xinxinyuanze222@outlook.com

**Keywords:** rice (*Oryza sativa* L.), high temperature, split potassium application, grain filling, endogenous hormones, grain quality

## Abstract

High temperature during flowering and grain filling severely reduces rice yield and grain quality. Split potassium (K) fertilization can mitigate such heat-induced damage, yet its mechanisms linking grain filling, endogenous hormones and grain performance remain unclear. Here, a two-year pot experiment was conducted to explore the effects of split K application on rice yield, quality and hormonal metabolism under high temperature. Four treatments included ambient temperature with full basal K (AT-K_100_), high temperature with full basal K (HT-K_100_), and two split K regimes under high temperature (HT-K_70+30_, HT-K_30+70_). Split K application decreased abscisic acid (ABA) levels at 5 days after anthesis (DAA), increased indole-3-acetic acid (IAA), zeatin riboside (ZR) and gibberellin A3 (GA_3_) at 5 DAA, and maintained higher IAA and GA_3_ levels until 20 DAA. The ratios of ABA/IAA and ABA/GA_3_ were also reduced at both 5 and 20 DAA. These hormonal alterations optimized grain-filling dynamics, prolonged active filling duration and improved middle- and late-stage filling rates, thereby promoting grain weight accumulation and suppressing chalkiness formation. Compared with HT-K_100_, HT-K_70+30_ increased yield by 8.75%, which was attributed to improved seed-setting rate and 1000-grain weight. HT-K_30+70_ enhanced spikelet number per panicle, seed-setting rate and 1000-grain weight, but significantly decreased effective panicles, resulting in no obvious yield advantage. Furthermore, split K application effectively reduced grain chalkiness, with a more pronounced effect at a higher panicle-stage K proportion. Under ongoing global warming, K management can be tailored to production goals: higher basal K is preferable for yield pursuit, while increasing panicle K topdressing effectively improves grain quality.

## 1. Introduction

Global warming has led to an increased frequency and duration of high-temperature events during summer in many regions worldwide [1]. For rice (*Oryza sativa* L.), the flowering and grain-filling stages are particularly sensitive to high-temperature stress. High temperature during this critical period poses a serious threat to both grain yield and quality [2]. Numerous studies have confirmed the negative impact of post-anthesis high-temperature stress on rice yield formation [3,4]. Concurrently, high temperature significantly exacerbates chalkiness—an undesirable grain trait that severely compromises milling, appearance, cooking, and eating qualities, thereby reducing market value [5,6,7]. Consequently, developing effective mitigation strategies is imperative to counter the challenges posed by a warming climate.

The grain-filling process, which dictates final grain weight and chalkiness, is highly sensitive to high-temperature stress, resulting in accelerated but abbreviated filling, lower grain weight [8,9] and higher chalkiness [10,11]. Grain filling in cereals is regulated by the source–sink relationship [12,13]. Multiple plant hormones, such as abscisic acid (ABA), cytokinins (CKs), indole-3-acetic acid (IAA), and gibberellins (GAs), are collectively involved in modulating sink activity in cereals [14,15]. ABA in grains promoted the translocation of photosynthates to developing grain and was positively correlated with grain-filling rate [16,17]. IAA played a regulatory role in stimulating endosperm cell division, while also facilitating cell expansion and assimilating translocation in rice grains [18]. ZR mediated cell division in rice endosperm, thereby regulating grain sink size [16,19]. GAs promoted endosperm cell development, extended the active grain-filling period, and improved grain plumpness [20]. Meanwhile, inhibition of GAs biosynthesis induced grain abortion and reduced grain weight [21]. As an adaptive strategy to environmental stress, plants actively modulate their endogenous hormone homeostasis. Generally, high temperature inhibited the accumulation of IAA, CKs and GAs [21,22,23,24,25], whereas it enhanced ABA biosynthesis and accumulation [21,22,23,24], thereby reshaping hormone balance and eventually altering grain-filling characteristics.

Nutrient management emerges as a highly effective strategy for enhancing crop resilience to temperature extremes [10,11,26]. Among essential nutrients, potassium (K) is particularly noteworthy due to its vital role in osmoregulation and its demonstrated efficacy in alleviating high-temperature stress [27]. K application has been shown to mitigate the adverse effects of high temperature on grain yield and quality [28,29,30,31]. Spray potassium dihydrogen phosphate (KH_2_PO_4_) during the grain-filling stage increased the average grain-filling rate and prolonged the duration of grain filling under high-temperature conditions, which resulted in a higher grain weight [32,33]. Moreover, K is known to modulate the distribution and transport of endogenous hormones in plants [34]. However, its influence on hormone profiles is complex and context-dependent. K application has been shown to enhance the concentrations of IAA, CKs, and GAs, while reducing ABA levels, in cotton under waterlogging stress [34] and in soybean seeds under non-stress conditions [35]. Conversely, under drought stress in wheat grains [36] or salt stress in the roots of *Tamarix ramosissima* [37], K application elevated the levels of IAA, CKs, and ABA. These contrasting responses highlight the complex and context-dependent nature of potassium’s influence on hormonal regulation. Notably, how K application modulates hormonal dynamics during grain filling to influence yield and chalkiness under rice-specific high-temperature stress remains poorly understood.

Rice exhibits a considerable requirement for K, which necessitates a sustained supply throughout the heading phase subsequent to the vegetative phase [38]. However, traditional smallholder farming practices often involve a single basal application of K fertilizer without subsequent top-dressing [39]. This practice can lead to leaching losses and result in K deficiency during the crucial heading and grain-filling stages. Previous studies have indicated that split application of K fertilizer can enhance rice yield [40,41] and grain quality [30,42]. Therefore, the primary objectives were: (i) to assess the influence of split K fertilizer application on rice yield and chalkiness under high-temperature conditions, (ii) to examine the correlation between grain weight, rice chalkiness, and the grain filling process, and (iii) to investigate the dynamic changes in endogenous hormones and their relationships with the grain-filling process, grain weight and rice chalkiness during the flowering and grain-filling stages under high-temperature conditions.

## 2. Materials and Methods

### 2.1. Experimental Design and Management

A pot experiment was undertaken in Jiaxing, Zhejiang Province, China (30°50′ N, 120°42′ E) during the years 2021 and 2022. Each plastic pot, measuring 27 cm in diameter and 33 cm in height, was filled with 15 kg of thoroughly mixed soil. The soil was purple clay paddy which contained 36.25 g kg^−1^ organic matter, 2.21 g kg^−1^ total nitrogen (N), 21.41 mg kg^−1^ available phosphorus (P), 56.36 mg kg^−1^ available potassium (K), 20.67 mg kg^−1^ ammonium-N (NH_4_–N), 7.52 mg kg^−1^ nitrate-N (NO_3_–N), and pH values of 6.49 in 2021, and 35.67 g kg^−1^ organic matter, 1.98 g kg^−1^ total N, 20.30 mg kg^−1^ available P, 53.73 mg kg^−1^ available K, 20.67 mg kg^−1^ and 17.02 mg kg^−1^ NH_4_–N, 7.08 mg kg^−1^ NO_3_–N, and pH values of 6.35 in 2022, respectively. Soil organic matter, total N, available K, and pH were determined as follows [43]: organic matter was analyzed using the potassium dichromate external heating oxidation method, total N was quantified with an AutoAnalyzer after Kjeldahl digestion and available K was measured by flame photometry, and pH was determined with a pH meter at a soil-to-water ratio of 1:2. Available P was quantified by the Mehlich 3 (M3) method [44]. NH_4_–N and NO_3_–N were extracted with KCl solution and determined using a continuous flow analyzer [45]. The locally predominant variety, Xiushui 134 (japonica rice), was selected for the experiment. Seeds were sown in prepared seedbeds on 25 May and 29 May in 2021 and 2022, respectively. Uniform seedlings were then transplanted into pots on 25 June and 29 June in 2021 and 2022, with one plant per hill and three hills per pot.

Plants grown in pots were initially exposed to ambient-temperature (AT) conditions before being transferred to a climate chamber with natural light for high-temperature (HT) treatment regimens, which commenced post-anthesis. Based on prior research [46,47] and considering the characteristics of natural high temperatures during summer in Jiaxing, two temperature treatments were established within the sunlit climate-controlled chambers: an optimal diurnal/nocturnal temperature of 28 °C/20 °C and a high diurnal/nocturnal temperature of 36 °C/28 °C. The temperatures in the climate chamber were designed to simulate the dynamic variations in natural conditions, with the lowest temperature occurring at 4:00 and the highest at 14:00 each day. Detailed temperature setting records are presented in Table 1. The HT treatment lasted for 20 days. A total of 40 pots were randomly assigned to the AT treatment, while 120 pots, within which three potassium (K) fertilizer management strategies—a conventional strategy (K_100_) applying K fertilizer exclusively as a basal dressing, and two split-application strategies allocating 70% of K before transplanting and 30% at the panicle initiation stage (K_70+30_), or 30% before transplanting and 70% at the panicle initiation stage (K_30+70_)—were randomly and equally applied to 40 pots each, were assigned to the HT treatment. The K_100_ management strategy was also applied to 40 pots in the AT treatment. Therefore, the study comprised four treatments: AT-K_100_, HT-K_100_, HT-K_70+30_, and HT-K_30+70_. K (K_2_O) fertilizer was applied at a rate of 0.8 g per pot, while phosphorus (P_2_O_5_) fertilizer was applied as a single basal dose of 0.6 g per pot. N was administered at a rate of 1.2 g per pot in a split application, with 50% applied pre-transplanting, 30% at the tillering stage, and 20% at the panicle initiation stage. N, P, and K were applied in the form of urea, calcium dihydrogen phosphate, and potassium chloride, respectively. All nutrients were supplied with analytical-grade reagents. Detailed fertilizer application regimes were presented in Table 2. Regarding water regimes, a 3–5 cm water layer was maintained from transplanting to late tillering. Soil moisture gradually decreased to 75–85% of field capacity to suppress unproductive tillers; then, a 2–3 cm shallow water layer was restored until booting. Alternate wetting and drying (AWD) was adopted during grain filling: the soil was flooded with 2–3 cm water and left to dry naturally to 85–95% soil moisture, with the cycle repeated throughout the stage. Soil moisture was maintained at 60–70% at maturity. Every three days, an ML2x sensor (Houston, TX, USA) and XK3100 electronic scale were used for gravimetric monitoring to ensure consistent soil moisture in all pots. All other management practices, including weed control and disease and pest management, were consistent across all experimental treatments.

### 2.2. Data Collection

At anthesis, fully developed panicles that bloomed synchronously were marked with plastic tags to document the flowering date. Commencing from 5 days after anthesis (DAA) and continuing until maturity, a sample of 30 tagged panicles was selected between 9:00 and 10:00 a.m. at 5-day intervals, and these panicles were evenly divided into three groups as three independent technical replicates. From each sampled panicle, grains (excluding hollow grains) were collected from four primary rachis branches located in the central region. Grain samples from each of the three replicates were dehulled and then randomly divided into two separate groups. One group was promptly frozen in liquid N and stored at an ultra-low temperature of −80 °C for subsequent hormone level assessments. The other group was initially dried at 105 °C for 30 min, followed by oven-drying at 70 °C until a constant mass was achieved. Following this, the grain weight was determined.

#### 2.2.1. Rice Yield and Its Components

Plants from three replicate pots per treatment were collected for the assessment of rice yield. The yield components, comprising the number of panicles per pot, spikelets per panicle, thousand-grain weight, seed-setting percentage, and spikelet fertility rate, were quantified using data from five pots. The 1000-grain weight was standardized to correspond with a moisture content of 14.5%. The spikelet fertility rate is defined as the proportion of fertile spikelets to the total number of spikelets.

#### 2.2.2. Rice Chalkiness

At maturity, a representative sampling of grains was conducted from four primary rachis branches positioned centrally within 80 randomly selected panicles per treatment. These samples were preserved at room temperature for a duration of three months to stabilize their characteristics. Following this period, the grains underwent dehusking via a ridge grain machine and were subsequently processed into milled rice using a rice milling machine. For each treatment, a subset of 100 head rice grains was randomly selected for the assessment of chalky grain rate and chalkiness area, and the selection and measurement were performed in three independent technical replicates. The determination of chalkiness area involved the use of a scanner (Epson Expression V700 Photo, Seiko Epson Co., Nagano, Japan) in conjunction with image analysis software version 1.54 (ImageJ, developed by the National Institutes of Health, Bethesda, MD, USA), adhering to the methodological guidelines established by Chen et al. [48]. The chalkiness degree was calculated as the product of the chalky grain incidence and the measured chalkiness area.

#### 2.2.3. Dynamics of Grain Filling

Richards’s growth equation [49,50] was adopted to delineate the process of grain filling. The computation of the characteristics pertaining to grain-filling was conducted as outlined below:

(1) $$W=\frac{A}{(1+Be^{-kt}{)}^{1/N}}$$

The determination of the grain-filling rate (G) was achieved through the computation of the derivative, as specified in Formula (1):

(2) $$G=\frac{AkBe^{-kt}}{N(1+Be^{-kt}{)}^{N+1/N}}$$

where *W* signifies the grain weight (mg), *A* denotes the final grain weight (mg), and *t* represents the days after anthesis. The parameters *B*, *k*, and *N* are coefficients derived from the regression equation.

The *R*_0_, *T_max_*, *D*, and *G_mean_* were derived using the respective formulas detailed here:

(3) $$R_{0}=\frac{k}{N}$$

(4) $$T_{max}=\frac{\ln B-\ln N}{k}$$

(5) $$D=\frac{2(N+2)}{k}$$

(6) $$G_{mean}=\frac{Ak}{2(N+2)}$$

where *R*_0_ stands for the initial grain-filling potential. *T_max_* is the time to reach a maximum grain-filling rate. *D*, the active grain-filling period, was defined as the duration when *W* ranged from 5% (*t*_1_) to 95% (*t*_2_) of *A*. Meanwhile, *G_mean_*, the mean grain-filling rate, was calculated as the average grain-filling rate from t_1_ to t_2_. By substituting *T_max_* into Formula (2), we can determine the maximum grain-filling rate, denoted as *G_max_*.

#### 2.2.4. Endogenous Hormones

ABA, IAA, ZR, and GA_3_ were extracted and purified from sample tissues, followed by quantitative determination via by liquid chromatography–tandem mass spectrometry [51]. For each treatment group, 0.1 g of grain samples was ground into a fine powder in liquid nitrogen and subsequently extracted 2 times with a total of 10 volumes of 80% (*v*/*v*) methanol containing 1% (*v*/*v*) acetic acid with internal standards, followed by overnight incubation at 4 °C. After centrifugation at 14,000 rpm and 4 °C for 10 min, the supernatant was collected. The supernatant was evaporated and redissolved in 1% (*v*/*v*) acetic acid aqueous solution, then loaded onto a pre-equilibrated Oasis HLB column cartridge (30 mg, 1 mL, Waters, Milford, MA, USA). The cartridge was rinsed with 1 mL of 1% (*v*/*v*) acetic acid aqueous solution, and hormones were eluted with 2 mL of 80% (*v*/*v*) acetonitrile containing 1% (*v*/*v*) acetic acid. The eluate was evaporated in vacuo to remove acetonitrile and reconstituted with 1% (*v*/*v*) acetic acid aqueous solution. The concentrated extract was further purified using a pre-equilibrated Oasis MCX column cartridge (30 mg, 1 mL, Waters). The MCX cartridge was washed with 1 mL of 1% (*v*/*v*) acetic acid aqueous solution, and the fraction containing IAA, GA_3_ and ABA was eluted with 2 mL of 80% (*v*/*v*) acetonitrile. An appropriate aliquot of the eluate was transferred, dried, and reconstituted with 1% (*v*/*v*) acetic acid aqueous solution for subsequent analysis. The MCX cartridge was further washed with 1 mL of 5% (*v*/*v*) aqueous ammonia, and the basic fraction containing ZR was eluted with 2 mL of 60% (*v*/*v*) acetonitrile containing 5% (*v*/*v*) aqueous ammonia. Acetonitrile was removed in vacuo before instrumental determination. Phytohormones were analyzed by LC-ESI-MS/MS using an Agilent 6470 triple quadrupole mass spectrometer. Chromatographic separation was performed on a ZORBAX Eclipse XDB-C18 column (Agilent). The analytical procedure followed the method described in reference [52]. Target quantification was conducted with MassHunter software (Version B.08.00, Agilent Technologies, Santa Clara, CA, USA).

### 2.3. Statistical Analysis

The data underwent statistical analysis utilizing either ANOVA or Pearson correlation tests implemented within SPSS version 27.0. Differences among the mean values of the various treatments were assessed using Tukey’s Honest Significant Difference (HSD) test, with a statistical significance level set at *p* < 0.05 and a highly significant level at *p* < 0.01. Microsoft Excel 2017 and OriginPro 2018 were employed for the purpose of data visualization.

## 3. Results

### 3.1. Rice Yield and Its Components

Analysis of variance revealed that the year (Y) had a significant effect on panicles per pot, 1000-grain weight and rice yield, whereas no significant difference was observed for the other yield component traits. Treatment (T) exhibited highly significant differences in grain yield and yield components of rice. In addition, the interaction between year and treatment exerted no significant influence on rice grain yield and its components. High temperature during the flowering and grain-filling stages significantly reduced rice yield by 17.58% (Table 3). This reduction was primarily due to an 11.96% decrease in the seed-setting percentage and a 6.24% decrease in the 1000-grain weight, whereas the numbers of effective panicles per plant and spikelets per panicle were not significantly affected. Under high-temperature conditions, the HT-K_70+30_ treatment increased yield by 8.75% compared to HT-K_100_, which was attributed to a 5.13% improvement in the seed-setting percentage and a 3.32% increase in the 1000-grain weight. Although the HT-K_30+70_ treatment significantly increased spikelets per panicle, seed-setting percentage, and 1000-grain weight by 4.81%, 7.98%, and 4.38%, respectively, it concurrently reduced the number of effective panicles per plant by 12.45%. Consequently, no significant yield difference was observed between the HT-K_30+70_ and HT-K_100_ treatments.

### 3.2. Rice Chalkiness

ANOVA analysis indicated that both Y and T significantly affected chalky grain rate and chalkiness degree. In contrast, their interaction exhibited no statistically significant effect on these two grain chalkiness traits (Table 4). Elevated temperature markedly increased both chalky grain rate and chalkiness degree. In 2021, relative to the AT-K_100_ treatment, the HT-K_100_ treatment increased the chalky grain rate by 138.46% and the chalkiness degree by 364.11%. A similar trend was observed in 2022: compared with the AT-K_100_ treatment, HT-K_100_ treatment caused the chalky grain rate to grow by 137.94% and the chalkiness degree to rise by 358.12%.

Compared with one-time basal K fertilization, split K application markedly lowered grain chalky rate and chalkiness degree. Under high-temperature stress, increasing the proportion of K fertilizer supplied at the heading stage further reduced these two grain chalkiness indices. Compared with the HT-K_100_ treatment, in 2021, the chalky grain rate and chalkiness degree of HT-K_70+30_ treatment dropped by 9.61% and 11.37%, while HT-K_30+70_ treatment decreased by 13.79% and 18.73%, respectively. In 2022, relative to HT-K_100_ treatment, HT-K_70+30_ treatment showed respective declines of 8.71% and 12.87% in chalky grain rate and chalkiness degree, and HT-K_30+70_ treatment exhibited reductions of 13.65% and 20.97%, respectively.

### 3.3. Grain-Filling Characteristics

**Figure 1:**
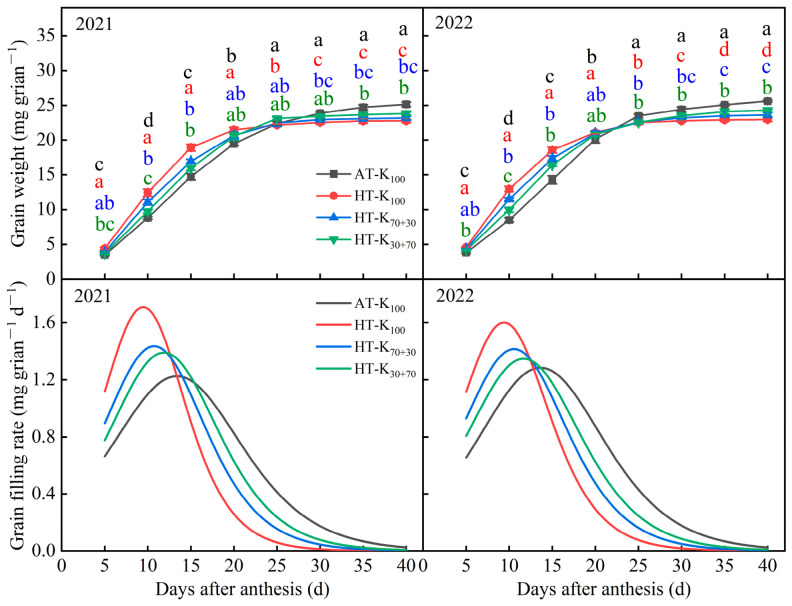
Grain weight and Richards simulation curve of grain filling of rice exposed to different K fertilizer management treatments under high temperatures. AT-K_100_, ambient temperature with basal K fertilizer; HT-K_100_, high temperature with basal K fertilizer; HT-K_70+30_, high temperature with the split application of 70% K before transplanting and 30% K at the panicle initiation stage; HT-K_30+70_, high temperature with the split application of 30% K before transplanting and 70% K at the panicle initiation stage. Data are presented as mean ± standard deviation (*n* = 3). Different lowercase letters indicate significant differences among treatments (*p* < 0.05).

The high determination coefficient (>0.99) of the Richards’ equation for grain filling (Table 5) indicates its suitability for simulating the grain-filling process. High temperature increased grain weight and grain-filling rate during the early grain-filling stage, along with elevated R_0_ and G_max_ (Figure 1). In contrast, during the later grain-filling stage, high temperature reduced grain weight and grain-filling rate, decreased G_mean_, advanced T_max_, and shortened D. Under high-temperature conditions, compared with HT-K_100_ treatment, both HT-K_70+30_ and HT-K_30+70_ treatments decreased grain weight and filling rate during the early grain-filling stage but increased them during the later grain-filling stage. Relative to HT-K_100_ treatment, HT-K_70+30_ treatment reduced R_0_, G_mean_, and G_max_ by an average of 3.98%, 9.76%, and 9.82%, respectively, while HT-K_30+70_ treatment reduced these parameters by 6.88%, 12.95%, and 13.02%, respectively. Moreover, HT-K_70+30_ treatment delayed T_max_ and D by an average of 0.8 and 2.8 days, respectively, and HT-K_30+70_ treatment delayed them by 1.7 and 4.5 days.

### 3.4. Hormonal Changes

**Figure 2:**
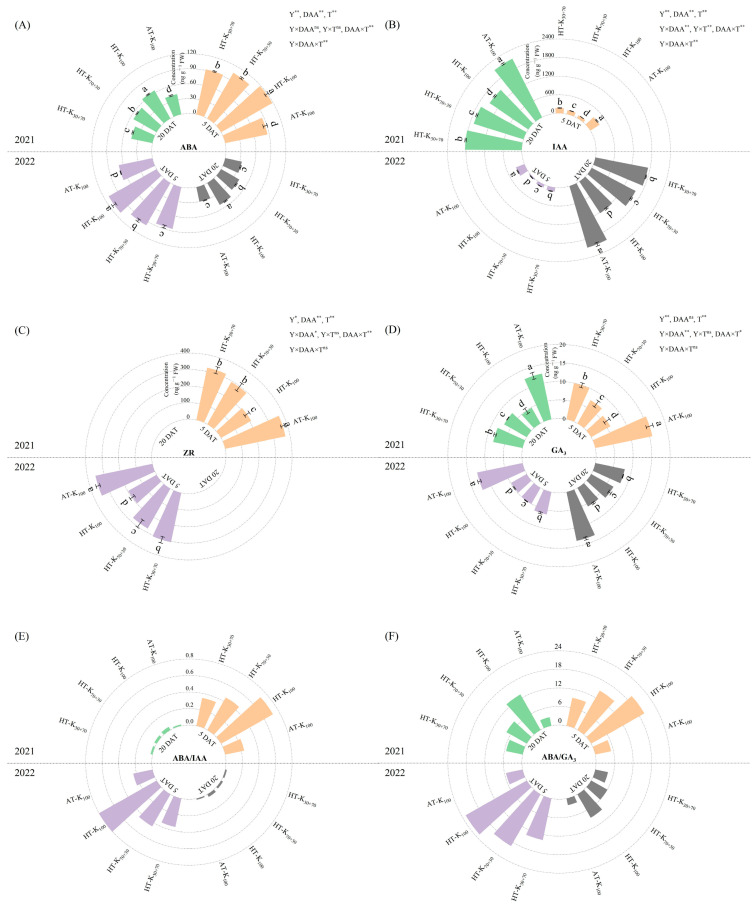
Endogenous hormones in grain exposed to different K fertilizer management treatments under high temperatures. (**A**) ABA; (**B**) IAA; (**C**) ZR; (**D**) GA_3_; (**E**) ABA/IAA; (**F**) ABA/GA_3_. AT-K_100_, ambient temperature with basal K fertilizer; HT-K_100_, high temperature with basal K fertilizer; HT-K_70+30_, high temperature with the split application of 70% K before transplanting and 30% K at the panicle initiation stage; HT-K_30+70_, high temperature with the split application of 30% K before transplanting and 70% K at the panicle initiation stage; Y, year; T, treatment; DAA, days after anthesis. * and ** signify statistical significance at the 0.05 and 0.01 probability level, and the ns denotes the absence of statistical significance at the 0.05 probability level for the observed mean differences. Data are presented as mean ± standard deviation (*n* = 3). Different lowercase letters indicate significant differences among treatments (*p* < 0.05).

ANOVA results indicated that ABA, IAA, ZR and GA_3_ exhibited differential responses to Y, DAA, T, and their interactive effects. ABA was significantly affected by Y, DAA, T, DAA × T and the three-way interaction Y × DAA × T, with no significant effects observed for Y × DAA and Y × T. All main factors, two-way interactions and the three-way interaction exerted significant influences on IAA. ZR responded significantly to Y, DAA, T, Y × DAA and DAA × T, while Y × T and Y × DAA × T had negligible effects. GA_3_ was significantly regulated by Y, T, Y × DAA and DAA × T, whereas DAA, Y × T and Y × DAA × T exerted no obvious effects (Figure 2). High-temperature stress induced distinct stage-dependent changes in endogenous hormone concentrations in grains. At both 5 and 20 DAA, HT-K_100_ treatment significantly increased ABA level, and the ratios of ABA/IAA and ABA/GA_3_ compared with AT-K_100_ treatment (*p* < 0.05). Conversely, HT-K_100_ treatment significantly reduced IAA, ZR and GA_3_ levels at 5 DAA, as well as IAA and GA_3_ levels at 20 DAA (*p* < 0.05). Split K application (HT-K_70+30_ and HT-K_30+70_ treatments) effectively rebalanced the high-temperature-disrupted hormone homeostasis. Specifically, compared with the conventional K application treatment (HT-K_100_), both HT-K_70+30_ and HT-K_30+70_ treatments significantly lowered ABA level and the ratios of ABA/IAA and ABA/GA_3_ at 5 and 20 DAA, whereas they increased IAA, ZR and GA_3_ levels at 5 DAA, and IAA and GA_3_ levels at 20 DAA (*p* < 0.05). ZR concentrations at 20 DAA may be extremely low and below the detection limit; thus, valid data were not available.

### 3.5. Correlation Between Seed Weight, Chalkiness and Grain-Filling Traits

**Figure 3:**
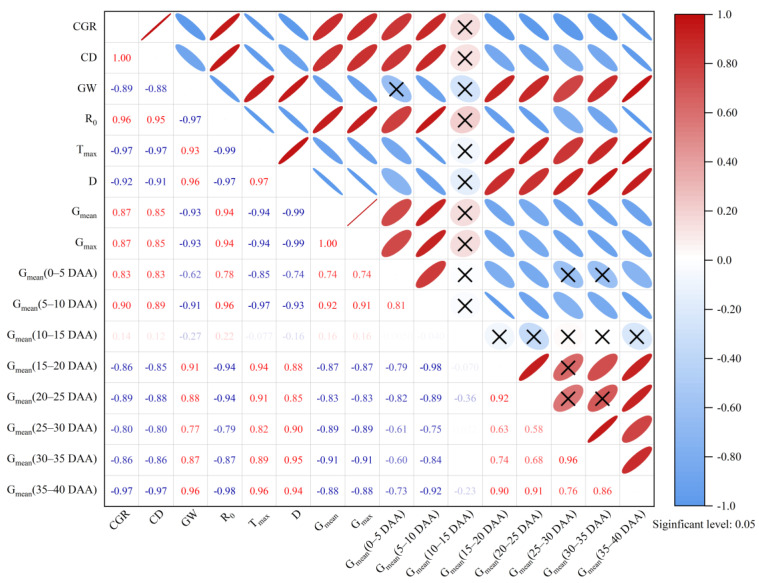
Correlations among rice chalkiness, seed weight, and grain-filling parameters. The more elongated and flatter the ellipse, the stronger the correlation between the two variables. Red ellipse represents negative correlations. Blue ellipse represents positive correlations. CGR, CD, and GW are chalky grain rate, chalkiness degree and seed weight. R_0_, initial grain growth potential; T_max_, the time to reach a maximum grain-filling rate. D, the active grain-filling period; G_mean_, the mean grain-filling rate; G_max_, the maximum grain-filling rate; DAA, days after anthesis. The black ✕ indicates no significant difference.

Pearson correlation analysis revealed significant associations among chalkiness-related traits (chalky grain rate and chalkiness degree), seed weight, and grain-filling parameters (Figure 3). Chalkiness traits were positively correlated with R_0_, G_mean_, G_max_, and the mean grain-filling rates during 0–5 and 5–10 DAA, but negatively correlated with T_max_, D, and the mean rates during 15–20, 20–25, 25–30, 30–35, and 35–40 DAA. No significant correlation was observed for the 10–15 DAA period. In contrast, seed weight exhibited an inverse correlation pattern: it was negatively correlated with the parameters positively associated with chalkiness (R_0_, G_mean_, G_max_, and the mean grain-filling rates during 0–5 and 5–10 DAA), and positively correlated with those negatively associated with chalkiness (T_max_, D, and the mean rates during 15–20, 20–25, 25–30, 30–35, and 35–40 DAA). These results indicate a coordinated relationship between chalkiness formation, seed weight accumulation, and grain-filling dynamics.

### 3.6. Correlation Between Grain-Filling Traits and Hormone Dynamics

**Figure 4:**
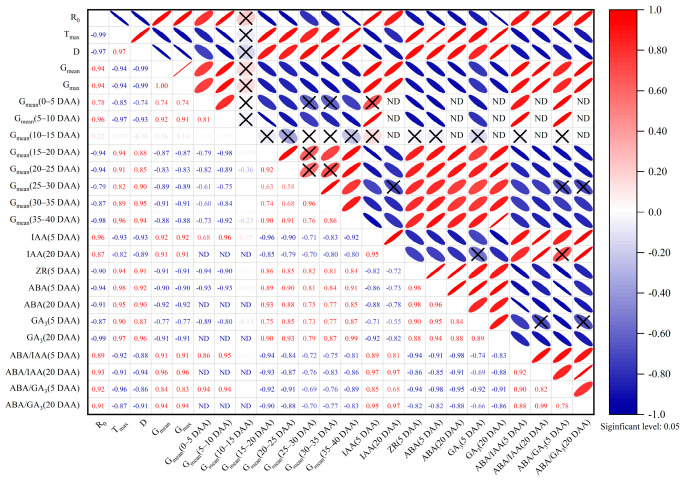
Relationship between grain-filling traits and endogenous hormones in grain. The more elongated and flatter the ellipse, the stronger the correlation between the two variables. Red ellipse represents negative correlations. Blue ellipse represents positive correlations. R_0_, initial grain growth potential; T_max_, the time to reach a maximum grain-filling rate. D, the active grain-filling period; G_mean_, the mean grain-filling rate; G_max_, the maximum grain-filling rate; DAA, days after anthesis. ABA, abscisic acid; IAA, indole-3-acetic acid; GA_3_, gibberellic acid; ZR, zeatin riboside. The black ✕ indicates no significant difference. Values marked ND indicate no biological significance, with relevant data not presented.

The Pearson correlation matrix revealed significant relationships between grain-filling parameters and endogenous hormone levels at key developmental stages under high-temperature and split K application conditions (Figure 4). At 5 DAA, ABA level was strongly positively correlated with R_0_, G_mean_, G_max_, and the mean grain-filling rates during 0–5 and 5–10 DAA, but negatively correlated with T_max_, D, and the mean grain filling rates during 15–20, 20–25, 25–30, 30–35, and 35–40 DAA. At 20 DAA, ABA level was strongly positively correlated with R_0_, G_mean_, and G_max_, but negatively correlated with T_max_, D, and the mean grain filling rates during 20–25, 25–30, 30–35, and 35–40 DAA. In contrast, at 5 DAA, the IAA, ZR and GA_3_ levels exhibited the opposite correlation pattern: negatively associated with the early parameters (R_0_, G_mean_, G_max_, and the mean grain filling rates during 0–5 and 5–10 DAA) and positively with the later parameters (T_max_, D, and the mean grain filling rates during 15–20, 20–25, 25–30, 30–35, and 35–40 DAA). At 20 DAA, the correlation pattern reversed: the IAA and GA_3_ levels showed strong positive correlations with T_max_, D, and the mean grain filling rates during 15–20, 20–25, 25–30, 30–35, and 35–40 DAA but negative correlations with R_0_, G_mean_, and G_max_. The ratios of ABA/IAA and ABA/GA_3_ showed correlation patterns with the grain-filling parameters consistent with that of ABA alone.

## 4. Discussion

High temperature during the flowering and grain-filling stage is a critical environmental stress factor that adversely affects rice yield and quality [53,54,55,56,57], and K management has been recognized as an effective strategy to mitigate such adverse effects [58,59,60]. Given the ameliorative effects of K fertilizer on the detrimental consequences of high temperatures, we deliberately applied K fertilizer at the panicle initiation stage to optimize its beneficial impacts during the flowering and grain-filling stage, with the aim of increasing grain yield and reducing rice chalkiness. The results of this study revealed that split K application could enhance tolerance to subsequent high temperatures of rice and alleviate the high-temperature-induced yield loss and chalkiness deterioration by optimizing grain-filling processes and hormonal balance, with distinct effects between different split ratios.

### 4.1. Responses of Grain Yield and Rice Chalkiness to High Temperatures and Split Application of K Fertilizer

Consistent with previous studies [4,61,62], high-temperature stress during the flowering and grain-filling stage significantly reduced rice yield, primarily attributed to the decreases in seed-setting rate and 1000-grain weight (Table 3). Notably, the number of effective panicles per plant and spikelets per panicle remained unaffected by high temperature (Table 3), as these traits had already been determined during the vegetative and early reproductive stages prior to the stress period (i.e., the flowering and grain-filling stage). Moreover, in the present study, high-temperature stress during the flowering and grain-filling stage significantly increased both the chalky grain rate and chalkiness degree of rice, which is consistent with the findings previously reported [54,55,63].

Numerous studies have demonstrated that K application could increase the number of effective panicles, spikelets per panicle, seed-setting rate and 1000-grain weight, thereby enhancing yield [64,65,66]. In the present study, K topdressing at panicle initiation stage (i.e., HT-K_70+30_ and HT-K_30+70_ treatments) significantly improved spikelets per panicle, seed-setting rate and 1000-grain weight compared with HT-K_100_, with these yield components increasing proportionally to the amount of K applied at the panicle initiation stage. This finding indicates that K supplementation at the panicle initiation stage is able to mitigate the adverse effects of high temperature on grain-filling performance. Notably, under high-temperature conditions, K management strategies exerted differential effects on the number of effective panicles. Compared with the one-time basal application of K fertilizer treatment (HT-K_100_), HT-K_70+30_ treatment (70% basal + 30% panicle initiation stage) showed a slight but non-significant reduction in effective panicles per plant, whereas HT-K_30+70_ treatment (30% basal + 70% panicle initiation stage) caused a significant decrease. Since K is well known to promote tiller emergence [67,68,69,70], insufficient K availability during the vegetative growth stage directly limits tiller establishment. This differential response could be attributed mainly to the varying distribution of K across treatments: the higher basal K in HT-K_70+30_ treatment partially sustained tiller initiation and spikelet differentiation in early growth, whereas the lower basal K in HT-K_30+70_ treatment failed to meet the demand for tiller formation, leading to a pronounced reduction in effective panicles. In addition, K applied at panicle initiation could enhance tolerance to subsequent high temperatures of rice and further enhance spikelet formation and grain filling, directly contributing to the increased spikelets per panicle, seed-setting rate, and 1000-grain weight observed in HT-K_70+30_ and HT-K_30+70_ treatments (Table 3). Ultimately, the final yield reflects a trade-off between panicle traits and grain-filling performance. HT-K_70+30_ treatment yielded more than HT-K_100_ treatment because the modest decline in panicle number was not only fully compensated but also overcompensated by significant increase in spikelets per panicle, seed-setting rate, and 1000-grain weight. In contrast, the significant loss of effective panicles in HT-K_30+70_ treatment counteracted the benefits of improved spikelets per panicle, seed-setting rate, and 1000-grain weight, resulting in no statistically significant yield advantage over HT-K_100_ treatment. Proper K fertilization practices are beneficial to the improvement of crop quality [59,60,71]. Beyond yield, the effect of split K application on grain chalkiness under high temperature showed distinct patterns (Table 4). Specifically, split K application (HT-K_70+30_ and HT-K_30+70_) effectively reduced grain chalkiness, and such an alleviation effect became more pronounced with the increase in K proportion applied at panicle initiation. Moreover, we noted noticeable interannual differences in rice yield and quality traits between the two growing seasons (Table 3 and Table 4). While temperature and water regimes were strictly maintained across years, natural light conditions inside the glass climate chambers inevitably differed. As reported in numerous previous studies, light conditions during grain filling strongly modulate grain development [72,73], which explains the year-to-year variation in the measured indices.

Collectively, the treatment with a higher proportion of K applied as basal fertilizer (70% basal + 30% at panicle initiation) produced the maximum grain yield. However, increasing the K proportion for topdressing at panicle initiation (30% basal + 70% at panicle initiation) achieved the greatest reduction in grain chalkiness. Under the ongoing trend of global warming, high-temperature events during rice reproductive stages are becoming increasingly frequent. These findings indicate that K application regimes can be rationally optimized to meet practical production needs under high-temperature stress during flowering and grain filling. For yield-oriented production, increased K allocation to basal fertilization is preferable. In contrast, appropriately elevating the proportion of K topdressing at panicle initiation is conducive to improving grain quality when high-quality rice is prioritized.

### 4.2. Responses of Grain Filling and Endogenous Hormones in Rice to Split Application of K Fertilizer Under High Temperature

Grain weight, a key determinant of rice yield, and grain chalkiness, a critical indicator affecting grain quality, are both closely intertwined with the grain-filling process [74]. In the present study, Pearson correlation analysis revealed that under high-temperature conditions during the flowering and grain-filling stage, grain weight was inversely correlated with the R_0_, the grain-filling rate before 10 DAA, G_max_, and G_mean_, while being positively correlated with T_max_, the grain-filling rate beyond 20 DAA and D (Figure 3). The relationship between grain chalkiness and these indicators is completely opposite to that between yield and these indicators (Figure 3). The grain chalkiness was positively correlated with the R_0_, the grain-filling rate before 10 DAA, G_max_, and G_mean_, while being negatively correlated with T_max_, the grain-filling rate beyond 20 DAA and D. It is a sign that an excessively high R_0_, along with accelerated grain-filling rates during the early grain-filling stage and an elevated G_mean_, failed to compensate for the loss in grain weight caused by the shortened D, thereby resulting in a reduction in grain weight. Furthermore, the sharp transition from a rapid to a slow grain-filling rate, coupled with the shortened D, led to insufficient starch accumulation in the grains and loose arrangement of starch granules, which in turn contributed to high grain chalkiness. Consistent with earlier reports [65,66], high temperature in this study increased R_0_ and the grain-filling rate during the early grain-filling stage (5–10 DAA), advanced T_max_, and reduced the grain-filling rate during the later grain-filling stage (after 20 DAA) while shortening D. The elevated early filling potential failed to offset the loss caused by slower grain-filling rate during the later grain-filling stage and a shortened active filling period, ultimately lowering grain weight. Moreover, our results showed that high temperature promoted early grain filling and shortened D, thereby elevating chalkiness degree (Table 4), in agreement with earlier studies [56,75].

The grain filling of cereals is governed by the source–sink relationship [12,13]. Plant hormones play a significant role in regulating the sink activity of cereal grain, with multiple hormonal signals collectively involved in this regulatory process [14,15]. Under high-temperature conditions during the flowering and grain-filling stages, hormonal homeostasis in grains is disrupted, which further affects both source and sink capacity, thereby impairing the grain filling process. Specifically, a higher ABA level in grains during the early grain-filling stage (Figure 4) enhances carbohydrate transport from the source to the sink, which in turn increases R_0_ and accelerates the grain filling rate during the early grain-filling stage (Table 5). However, this enhanced carbohydrate translocation creates intense competitive pressure for assimilates derived from source organs, accelerating the remobilization of photoassimilates stored in leaf tissues [76]. This excessive remobilization leads to rapid carbohydrate depletion in source organs, which directly weakens source strength and triggers premature leaf senescence, thereby impairing overall source capacity [77]. On the other hand, endosperm cell division and expansion during the early stage are critical for sink capacity, which ultimately determines the final grain size [78]. Lower IAA, ZR and GA_3_ levels at 5 DAA (Figure 2) inhibited endosperm cell division and then decreased potential sink capacity [79,80,81]. Moreover, IAA and GA_3_ exhibit antagonistic effects with ABA [82,83], among which ABA has the function of accelerating seed maturation [84]. When the ratio of ABA/IAA and ABA/GA_3_ was higher, IAA and GA_3_ cannot effectively antagonize ABA-induced seed maturation, thereby causing premature termination of grain filling and earlier ripening. Furthermore, lower IAA and GA_3_ levels in grains at 20 DAA likely weaken the translocation of photosynthates to the grain sink. Together, these responses impaired sink activity. Thus, high temperature created a combined source–sink limitation during the middle and later grain-filling stages, which reduced the grain-filling rate at the middle and late stages and shorted the effective grain-filling duration.

K application effectively modulated this hormonal imbalance. Consistent with previous reports [34,85,86,87,88], split K application (HT-K_70+30_ and HT-K_30+70_) inhibited ABA accumulation and raised IAA and GA_3_ levels at 5 and 20 DAA, while it elevated ZR level at 5 DAA. Previous studies have demonstrated that K application alleviates the damage induced by high-temperature stress on the leaf photosynthetic system and improves the synthesis and supply capacity of photosynthates [89]. Meanwhile, the HT-K_70+30_ and HT-K_30+70_ treatments down-regulated ABA level at 5 DAA in grains (Figure 3), which prevents the excessively fast translocation of photosynthates to grain [90]. This regulation not only lowered R_0_ and the grain-filling rate during the early grain-filling stage under high-temperature conditions (Table 5), but also reduced the excessive remobilization of leaf reserves, and thereby prevented premature leaf senescence [91,92] and eventually improved source strength. The HT-K_70+30_ and HT-K_30+70_ treatments significantly increased the IAA, ZR and GA_3_ levels in grains at 5 DAA, which promoted endosperm cell division and improved the potential sink capacity. These treatments also elevated IAA and GA_3_ levels in grains at 20 DAA, effectively promoting the translocation of photosynthates to grains during the late grain-filling period. In addition, these treatments decreased the ratios of ABA/IAA and ABA/GA_3_ in grains at 5 and 20 DAA, effectively alleviating the premature maturation of grains. Consequently, such hormonal regulation increased the grain-filling rate at the middle and late stages and prolonged the effective grain-filling duration (Table 5). A relatively high grain-filling rate during the later stage and prolonged grain-filling duration can well compensate for the grain losses caused by a relatively low grain-filling rate during the early stage under HT-K_70+30_ and HT-K_30+70_ treatments, thereby increasing grain weight relative to HT-K_100_ treatment. In addition, higher GA3 level at 5 and 20 DAA under HT-K_70+30_ and HT-K_30+70_ treatments improved the seed-setting rate (Table 3). A previous study showed that a steady grain-filling rate is required for the prevention of chalkiness in rice endosperm [9,93]. HT-K_70+30_ and HT-K_30+70_ treatments exhibited a relatively smooth and steady grain-filling course and then help to reduce the formation of chalkiness.

It is notable that split K fertilization could strengthen rice thermotolerance to subsequent high temperature and that it alleviated the adverse effects of high temperature on rice yield and grain chalkiness; however, its effectiveness in mitigating these adverse impacts remains constrained relative to the degree of rice yield loss and chalkiness increase caused by high temperature. Since crop tolerance to high-temperature stress is jointly determined by genetic factors and cultivation practices, future research should integrate the breeding of heat-resistant rice varieties with the optimization of cultivation measures. This integrated approach is essential to further mitigate the detrimental impacts of high temperatures on rice yield and quality. Additionally, the spatial constraints of the climate chamber restricted the present experiment in two key aspects: first, only a single japonica rice cultivar was employed; second, the study was limited to testing three distinct split application strategies for K fertilizer under high-temperature conditions. Given these limitations, subsequent studies are required to identify the optimal split application ratio of K fertilizer in the context of global warming. Moreover, to verify the universal applicability of split K fertilizer application in alleviating high-temperature-induced reductions in rice yield and degradation of rice quality, it is necessary to expand the research scope to include different types of rice cultivars.

## 5. Conclusions

By enhancing rice thermotolerance, split K fertilization maintains stable grain filling under high temperature and alleviates heat-induced declines in grain yield and increases in grain chalkiness. This beneficial effect is primarily driven by the modulation of endogenous hormone homeostasis, via (1) reducing ABA level during the early grain-filling stage to avoid excessive early grain filling, (2) increasing IAA, ZR and GA_3_ during the early grain-filling stage to expand sink capacity, and (3) sustaining higher IAA and GA_3_ levels and lower ratios of ABA/IAA and ABA/GA_3_ during the middle-later grain-filling stage to promote late grain filling and prolong filling duration. Collectively, these adjustments yielded a smooth, stable grain-filling process, which in turn suppressed grain chalkiness formation. Furthermore, the high grain-filling rate during later grain-filling stage and prolonged duration of effective grain-filling compensated for grain weight losses from low grain-filling rate during the early grain-filling stage, ultimately boosting grain weight. The two split K application ratio treatments exhibited differences in their effectiveness in alleviating high-temperature-induced damage to rice, regarding grain chalkiness and yield. With global warming progressing, rice is increasingly threatened by heat events across the reproductive period. To better meet field production requirements, K fertilization strategies should be rationally adjusted according to specific cultivation objectives. For yield-oriented production, a larger share of K is recommended for basal application. In contrast, when grain quality is the primary production target, appropriately increasing K topdressing at panicle initiation can effectively improve rice quality.

## Figures and Tables

**Table 1:** Temperature setting of climate chamber during flowering and grain-filling stage.

Time	Temperature Setting of Different Temperature Treatments (°C)	
	Ambient-Temperature (AT)	High-Temperature (HT)
0:00–1:00	23.0	30.0
1:00–2:00	22.0	29.0
2:00–3:00	21.0	28.5
3:00–4:00	20.0	28.0
4:00–5:00	20.5	28.5
5:00–6:00	21.0	29.0
6:00–7:00	21.5	29.5
7:00–8:00	22.0	30.0
8:00–9:00	23.0	31.0
9:00–10:00	24.0	32.0
10:00–11:00	25.0	33.0
11:00–12:00	26.0	34.0
12:00–13:00	27.0	35.0
13:00–14:00	28.0	36.0
14:00–15:00	28.5	35.5
15:00–16:00	28.0	35.0
16:00–17:00	27.5	34.5
17:00–18:00	27.0	34.0
18:00–19:00	26.5	33.5
19:00–20:00	26.0	33.0
20:00–21:00	25.5	32.5
21:00–22:00	25.0	32.0
22:00–23:00	24.5	31.5
23:00–24:00	24.0	31.0

**Table 2:** Fertilizer application details of experimental treatments.

Treatments	Base Fertilizer (g)			Tiller Fertilizer (g)	Panicle Fertilizer (g)	
	N	P_2_O_5_	K_2_O	N	N	K_2_O
AT-K_100_	0.60	0.60	0.80	0.36	0.24	0.00
HT-K_100_	0.60	0.60	0.80	0.36	0.24	0.00
HT-K_70+30_	0.60	0.60	0.56	0.36	0.24	0.24
HT-K_30+70_	0.60	0.60	0.24	0.36	0.24	0.56

AT-K_100_, ambient temperature with basal K fertilizer; HT-K_100_, high temperature with basal K fertilizer; HT-K_70+30_, high temperature with the split application of 70% K before transplanting and 30% K at the panicle initiation stage; HT-K_30+70_, high temperature with the split application of 30% K before transplanting and 70% K at the panicle initiation stage. All fertilizers were individually dissolved in water, and corresponding volumes of the prepared solutions were added to the soil.

**Table 3:** Yield and its components of rice exposed to different K fertilizer management treatments under high temperatures.

Year	Treatment	Panicles per Pot	Spikelets per Panicle	Seed-Setting Rate (%)	1000-Grain Weight (g)	Yield (g Pot^−1^)
2021	AT-K_100_	28.0 ±1.00 a	130.7 ± 1.53 bc	89.79 ± 0.43 a	25.26 ± 0.18 a	82.99 ± 2.97 a
	HT-K_100_	28.3 ± 0.58 a	129.3 ± 0.58 c	80.67 ± 0.85 d	22.90 ± 0.34 c	67.68 ± 0.26 c
	HT-K_70+30_	27.7 ± 0.58 a	134.0 ± 2.00 ab	83.58 ± 0.89 c	23.56 ± 0.05 b	72.98 ± 0.53 b
	HT-K_30+70_	24.7 ± 0.58 b	136.3 ± 1.53 a	86.06 ± 0.15 b	23.90 ± 0.07 b	69.17 ± 0.73 bc
2022	AT-K_100_	25.7 ± 0.58 a	129.3 ± 1.53 b	92.26 ± 1.63 a	26.05 ± 0.23 a	79.98 ± 2.62 a
	HT-K_100_	26.0 ± 1.00 a	128.7 ± 1.53 b	81.60 ± 1.40 c	23.37 ± 0.24 c	63.79 ± 2.46 c
	HT-K_70+30_	25.3 ± 0.58 a	135.3 ± 1.15 a	85.21 ± 1.59 bc	24.19 ± 0.10 b	70.64 ± 0.90 b
	HT-K_30+70_	22.3 ±0.58 b	138.7 ± 1.15 a	86.29 ± 0.82 b	24.50 ± 0.08 b	65.45 ± 1.15 c
Analysis of Variance	Y	**	ns	ns	**	**
	T	**	**	**	**	**
	Y × T	ns	ns	ns	ns	ns

AT-K_100_, ambient temperature with basal K fertilizer; HT-K_100_, high temperature with basal K fertilizer; HT-K_70+30_, high temperature with the split application of 70% K before transplanting and 30% K at the panicle initiation stage; HT-K_30+70_, high temperature with the split application of 30% K before transplanting and 70% K at the panicle initiation stage; Y, year; T, treatment. ** signify statistical significance at the 0.01 probability level, and the ns denotes the absence of statistical significance at the 0.05 probability level for the observed mean differences. Data are presented as mean ± standard deviation (*n* = 3). Different lowercase letters indicate significant differences among treatments (*p* < 0.05).

**Table 4:** Grain chalkiness exposed to different K fertilizer management treatments under high temperatures.

Year	Treatment	Chalky Grain Rate (%)	Chalkiness Degree (%)
2021	AT-K_100_	25.93 ± 0.95 d	5.27 ± 0.58 d
	HT-K_100_	61.50 ± 0.53 a	24.10 ± 1.13 a
	HT-K_70+30_	55.13 ± 1.45 b	21.30 ± 0.79 b
	HT-K_30+70_	50.30 ± 1.14 c	18.30 ± 0.53 c
2022	AT-K_100_	26.43 ± 1.15 d	5.77 ± 0.50 d
	HT-K_100_	67.57 ± 1.27 a	27.23 ± 1.27 a
	HT-K_70+30_	59.57 ± 1.19 b	24.67 ± 0.76 b
	HT-K_30+70_	53.80 ± 1.78 c	20.13 ± 0.76 c
Analysis of Variance	Y	**	**
	T	**	**
	Y × T	ns	ns

AT-K_100_, ambient temperature with basal K fertilizer; HT-K_100_, high temperature with basal K fertilizer; HT-K_70+30_, high temperature with the split application of 70% K before transplanting and 30% K at the panicle initiation stage; HT-K_30+70_, high temperature with the split application of 30% K before transplanting and 70% K at the panicle initiation stage; Y, year; T, treatment. ** signify statistical significance at the 0.01 probability level, and the ns denotes the absence of statistical significance at the 0.05 probability level for the observed mean differences. Data are presented as mean ± standard deviation (*n* = 3). Different lowercase letters indicate significant differences among treatments (*p* < 0.05). In the table, higher chalky grain rate and chalkiness degree represent poorer grain quality.

**Table 5:** Parameters of grain-filling characteristics exposed to different K fertilizer management treatments under high temperatures.

Year	Treatment	Parameter									
		*A*	*B*	*k*	*N*	*R* ^2^	*R*_0_	*T_max_*	*D*	*G_mean_*	*G_max_*
2021	AT-K_100_	25.51	1.43	0.15	0.26	0.997	0.563	11.5	30.6	0.83	1.23
	HT-K_100_	22.75	2.49	0.23	0.35	0.999	0.657	8.4	20.3	1.12	1.66
	HT-K_70+30_	23.20	2.08	0.20	0.32	0.995	0.627	9.2	23.0	1.01	1.49
	HT-K_30+70_	23.90	2.20	0.19	0.32	0.995	0.608	10.0	23.9	1.00	1.48
2022	AT-K_100_	25.90	1.64	0.15	0.28	0.997	0.550	11.5	29.6	0.87	1.29
	HT-K_100_	22.95	2.12	0.22	0.34	0.999	0.646	8.3	21.2	1.08	1.60
	HT-K_70+30_	23.66	1.73	0.19	0.31	0.996	0.624	9.1	24.1	0.98	1.45
	HT-K_30+70_	24.42	1.59	0.17	0.28	0.997	0.606	10.0	26.6	0.92	1.36

AT-K_100_, ambient temperature with basal K fertilizer; HT-K_100_, high temperature with basal K fertilizer; HT-K_70+30_, high temperature with the split application of 70% K before transplanting and 30% K at the panicle initiation stage; HT-K_30+70_, high temperature with the split application of 30% K before transplanting and 70% K at the panicle initiation stage; *A*, *B*, *k*, and *N* represent the parameters defined by the regression equation, with *R*^2^ denoting the model’s goodness of fit. *T_max_* refers to the time to reach a maximum grain-filling rate, G_max_ stands for the maximum grain-filling rate, *G_mean_* denotes the mean grain-filling rate, and *D* represents the active grain-filling period.

## Data Availability

The original contributions presented in this study are included in the article. Further inquiries can be directed to the corresponding author.

## Abbreviations

AT-K_100_, ambient temperature with basal K fertilizer; HT-K_100_, high temperature with basal K fertilizer; HT-K_70+30_, high temperature with the split application of 70% K before transplanting and 30% K at the panicle initiation stage; HT-K_30+70_, high temperature with the split application of 30% K before transplanting and 70% K at the panicle initiation stage; R0, initial grain growth potential; Tmax, the time to reach a maximum grain-filling rate. D, the active grain-filling period; Gmean, the mean grain-filling rate; Gmax, the maximum grain-filling rate; DAA, days after anthesis. ABA, abscisic acid; IAA, indole-3-acetic acid; GA3, gibberellin A3; ZR, zeatin riboside.
